# Relationship of Neuropeptide S with Clinical and Metabolic Parameters of Patients during Rehabilitation Therapy for Schizophrenia

**DOI:** 10.3390/brainsci12060768

**Published:** 2022-06-11

**Authors:** Agnieszka Markiewicz-Gospodarek, Renata Markiewicz, Beata Dobrowolska, Ryszard Maciejewski, Bartosz Łoza

**Affiliations:** 1 Department of Human Anatomy, Medical University of Lublin, 20-090 Lublin, Poland; agnieszkamarkiewiczgospodarek@umlub.pl (A.M.-G.); renata.markiewicz@umlub.pl (R.M.); 2 Department of Neurology, Neurological and Psychiatric Nursing, Medical University of Lublin, 20-093 Lublin, Poland; ryszard.maciejewski@umlub.pl; 3 Department of Holistic Care and Management in Nursing, Medical University of Lublin, 20-081 Lublin, Poland; 4 Department of Psychiatry, Medical University of Warsaw, 02-091 Warsaw, Poland; bartosz.loza.med@gmail.com

**Keywords:** neuropeptide S, PANSS, schizophrenia, rehabilitation

## Abstract

Neuropeptide S (NPS) is a factor associated with the central regulation of body weight, stress, anxiety, learning, memory consolidation, wakefulness–sleep cycle, and anti-inflammatory and neuroplastic effects. Its stress-reducing, anti-anxiety, arousal without anxiety, and pro-cognitive effects represent an interesting option for the treatment of neuropsychiatric disorders. The purpose of the study was to examine the potential associations of NPS levels in the blood with clinical and metabolic parameters during the rehabilitation therapy of patients with schizophrenia. Thirty-three male subjects diagnosed with schizophrenia were randomly divided into two groups. The rehabilitation group (REH, N16) consisted of patients who were subjected to structured, 3-month intensive rehabilitation therapy, and the control group (CON, N17) consisted of patients who were subjected to a standard support mechanism. Both groups continued their pharmacological treatment as usual. The NPS concentration, as well as clinical and metabolic parameters, were compared in both groups. Additionally, a group of healthy (H) males (N15) was tested for NPS reference scores. To look for the specificity and selectivity of the NPS relationship with clinical results, various factor models of the positive and negative syndrome scale (PANSS) were analyzed, including the original PANSS 2/3 model, its modified four-factor version, the male-specific four-factor model, and two five-factorial models validated in large groups in clinical and multi-ethnic studies. Results and conclusions: (1) Structured rehabilitation therapy, compared to unstructured supportive therapy, significantly reduced the level of schizophrenia disorders defined by various factor models derived from PANSS. (2) The clinical improvement within the 3-month rehabilitation therapy course was correlated with a significant decrease in neuropeptide S (NPS) serum level. (3) The excitement/Hostility (E/H) factor, which included schizophrenic symptoms of the psychotic disorganization, was specific and selective for the reduction in serum NPS, which was stable across all analyzed factor models. (4) The long-term relationship between serum NPS and clinical factors was not accompanied by basic metabolic parameters.

## 1. Introduction

Neuropeptide S (NPS) is a 20-aminoacid ligand, with the name originating from the Serine N-terminal, found in human beings and nearly all tetrapods [1,2]. NPS is bound specifically to the G-protein-coupled receptor (NPSR1), which stimulates the intracellular Ca^2+^ and cAMP signaling. The NPS peptide precursor mRNA is found only in limited regions of the brain (trigeminal nucleus, lateral parabrachial nucleus, locus coeruleus, and amygdala), and in contrast, NPSR1 mRNA is widely expressed in the entire central nervous system (CNS) [2,3]. NPS fibers project to limbic and thalamic areas such as the amygdala, hypothalamus, and paraventricular thalamic nucleus [4]. In humans, the distribution of NPS and NPSR1 mRNA-expressing neurons was mainly found in the regions of importance for the integration of autonomic information and emotional behavior, such as the parabrachial area [5].

The main task of the NPS is the signaling and modulatory function of various emotional states (including fear and anxiety), which is associated with the activity of the HPA axis. Not only neuropeptides, but also neurotransmitters, hormones, and cytokines are involved in the transmission of all nerve impulses. The difference in functions between neuropeptides (including NPS) and neurotransmitters lies in their different activity, response, and target site of action. The effect of neuropeptides is slow, but also much stronger, resulting in an apparent change in the modulation of the regulatory mechanism of metabolic pathways and gene expression. Markiewicz et al. described the pharmacokinetic mechanism in detail in a previous publication [6].

Preclinical and clinical studies of the NPS/NPSR1 system have remained separated thus far, and there is no comprehensive description of the role of this system in either humans or rodents [2]. The NPS/NPSR1 system seems to play a significant role in stress responsiveness and the activation of the hypothalamic–pituitary–adrenal axis in rodents [2,7]. NPS activity is associated with inhibitory neurons that gate the amygdala output [8]. The NPS/NPSR1 system also participates in the regulation of the wakefulness–sleep cycle [9]. It is, therefore, assumed that since the NPS metabolism is highly conservative across different species, research on animals may be relatively well extrapolated to humans [8]. While such assumptions can be true in the case of the behavioral regulation of anxiety [1], arousal [1], or pain [8,10], it is difficult to simply extrapolate this way with the assumed role of NPS/NPSR1 in drug addiction [11,12], memory consolidation, and conceptual generalization [12], or even personality formation [11].

The NPS/NPSR1 system is also related to peripheral activity, e.g., immunological responses in asthma, rhinoconjunctivitis, rheumatoid arthritis, inflammatory bowel disease, enteral dysmotility, and permeability [9,13,14]. The role of NPS/NPSR1 in food intake is not clear. The anorexigenic effect of NPS was demonstrated in CNS animal studies; however, once injected through the orexin system, it could show a rebound effect [15]. The specific NPSR1 polymorphism was revealed in obese males, but a lower concentration of NPS was recorded independent of genotype in obesity [16].

The therapeutic use of the NPS/NPSR1 system in humans was suggested from the very beginning of the discovery of NPS [1]. NPS/NPSR1 activity could potentially be useful in the therapy of various anxiety disorders [17]. The authors of animal studies predicted that the NPS/NPSR1 system would facilitate the extinction of conditioned fear [2,18]. Specifically, the anxiolytic effect is not related to excessive sedation, but rather to an increase in activity (“novel activating anxiolytic”), which is a pharmacologically unique feature [17]. The median plasma NPS level was found to be significantly higher in generalized anxiety disorder (GAD) patients [19]. While NPS may have a beneficial effect on anxiety, no direct effect on depression has yet been demonstrated in animal models [20]. The NPS/NPSR1 system could be the platform for drug development on wakefulness–sleep disorders [8], to alleviate motor and non-motor dysfunctions of Parkinsonian diseases [3], to improve learning and memory, e.g., in Alzheimer’s disease [21], and to treat substance abuse disorders [22,23].

There are only preliminary data on the relationship between the NPS/NPSR1 system and the course of schizophrenia. A case–control comparison revealed that the low-functioning NPSR1 Asn107 variant was significantly associated with schizophrenia [24]. However, another study revealed no genetic association of NPSR1 alleles with schizophrenia (and ADHD), suggesting a rather specific relationship of NPSR1 with anxiety disorders [25]. There are various separate animal patterns for specific dysfunctions that could support the diagnostic and/or therapeutic potential of the NPS/NPSR1 system in schizophrenia research, for example, the “acoustic startle response” [24], but there is no comprehensive animal model to directly transfer these data to human pre-clinical or clinical models. The crux of the psychopharmacological effect of NPS on schizophrenia psychopathology may be due to the blocking of NMDA antagonist-induced deficits in pre-pulse inhibition [24,25,26]. NPS blocks MK-810 NMDA antagonism, suggesting a potential antipsychotic effect of NPS, such as MK-801, which blocks NMDA transmission and serves as a pharmacological model of schizophrenia [25,26]. Nevertheless, the similarity of NPS to anti-psychotics is not complete as haloperidol and sulpiride, both dopamine D2 receptor antagonists, which inhibit NPS-induced anti-nociceptive activity [9]. Long-term olanzapine administration led to the upregulation of NPS and downregulation of NPSR expression in the rat hypothalamus [27]. Chronic haloperidol administration led to the upregulation of NPS and NPSR in the rat brainstem [28]. These animal results suggest that anti-psychotics may work by affecting peptidergic signaling; however, they do not provide answers about the real impact of the NPS/NPSR1 system on schizophrenia.

The impact of intensive rehabilitation, especially with the use of the neurofeedback (NF) technique, on the level of peptide factors such as BDNF and the relationship with the clinical state, has already been shown in human studies [29,30]. However, no studies on the relationship between plasma NPS in patients with schizophrenia and any type of treatment have been published thus far. Although investigations of NPS’s permeability from the blood–brain barrier have not been conducted on human subjects, the rationality of measuring the plasma NPS level in patients with mental disorders has been demonstrated [19]. The authors of this study, while analyzing previous research supported by clinical knowledge, assumed that since the main function of NPS is a regulatory–modulatory mechanism, there is a probability of obtaining a positive clinical effect under the influence of rehabilitative interventions in a group of people with diagnosed schizophrenia. The assumption that cognitive functions improve along with a decrease in the level of NPS was the main objective of this study. Many publications and literature items emphasize the negative impact of stress on cognitive processes and, thus, on social cognition [6]. This approach is supported by the fact that neuropeptides perform functions analogous to those performed by neurotransmitters, neuromodulators, and neurohormones [31]. The plasma NPS levels could enable the identification of GAD with clinically useful specificity and sensitivity.

The aim of the study was to examine the potential associations of levels of NPS in the blood with clinical and metabolic parameters during the rehabilitation therapy of patients with schizophrenia. The starting point for the research was the assumption that the rehabilitation interventions had an impact on social cognition in sick people, which reflects complex psychological processes related to the reception of information, its coding, processing, and retrieval. Since these processes reflect the level of patients’ functioning, about a conclusion can be derived about the intensity of disturbances in these dimensions, and, thus, about the level of social activity or interpersonal relations. Although the definition of social cognition is imprecise, the basics of neurobiology in this area are considered a priority in treatment and rehabilitation [32]. Therefore, two main hypotheses were adopted in the study: (1) structured rehabilitation can reduce the level of NPS in the blood serum and (2) can affect the clinical improvement of the mental state of the subjects and biochemical indicators.

## 2. Materials and Methods

### 2.1. Study Design

This study was a randomized, controlled, 3-month trial reported with the use of Consolidated Standards of Reporting Trials (CONSORT) guidelines [33]. The trial was registered in the ISRCTN registry (trial ID: ISRCTN78612833), where the full protocol can be found.

Thirty-three male patients with paranoid schizophrenia (according to ICD-10-DCR [34]) were divided into two groups: a group in an intensive rehabilitation program (REH, N16), and a control group with standard social support (CON, N17). Since we planned the analysis of one independent variable at a given moment of time, a safe rule of thumb would be a minimum sample size of 2 × 15 (CON, REH). Members of both groups were recruited from the participants of a city day-care center program. They continued their anti-psychotic treatment and usual clinical management. Additionally, a group of healthy (H), non-clinical males (N15) with comparable characteristics was considered to check NPS reference results.

### 2.2. Participants

The inclusion criteria (CON and REH groups) included patients’ consent, male gender, clinical diagnosis of paranoid schizophrenia [34], age 18–50, right-handedness (writing), no current neurological diseases, mental disability, or alcohol and/or psychoactive substance addiction. The inclusion criteria in the non-clinical group (H) were the same as above, but they were all mentally healthy men. The study was limited only to male participants to reduce the risk of potential gender differences in NPS levels, which could not be corrected reliably between relatively small groups. Previous NPS studies with a limited number of participants clearly indicated difficulties in interpreting the results in relation to gender [19,20,25]. Moreover, PANSS results can also be influenced by gender differences [35].

The subjects, after the inclusion criteria were fulfilled, were assigned to two groups (CON, REH), with the allocation to the groups being random (drawing), without the researchers participating in the drawing process and without affecting the result.

All recruited patients had remained relatively stable, i.e., without active psychotic episodes for no less than 18 months. Despite the general difficulties in differentiating the types of schizophrenia, the patients could not be treated as clinically “residual” according to ICD-10-DCR, as they were quite young and active, multi-episodic, so they fit the pattern of episodic schizophrenia more closely, with stable or progressive development of negative symptoms in the intervals between psychotic episodes (ICD-10-DCR: F20.01/F20.02) [34,36]. No current suicidal risk was diagnosed.

**Figure 1:**
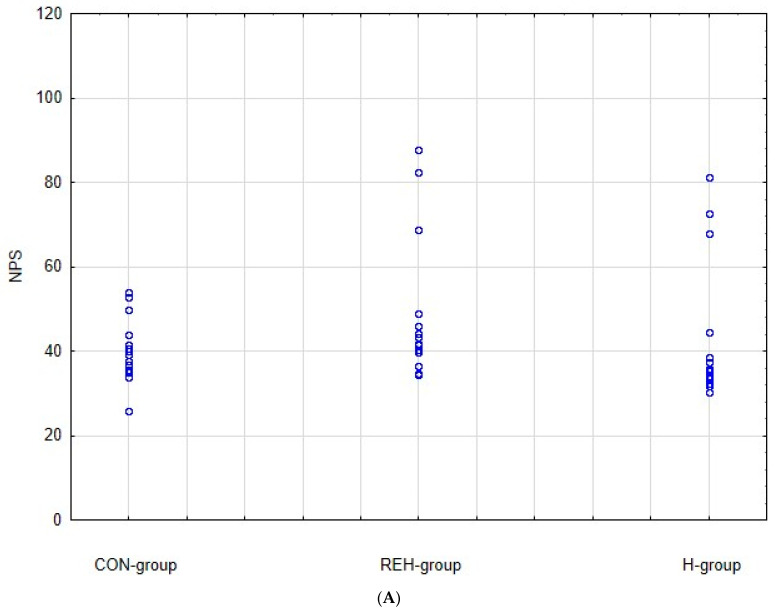

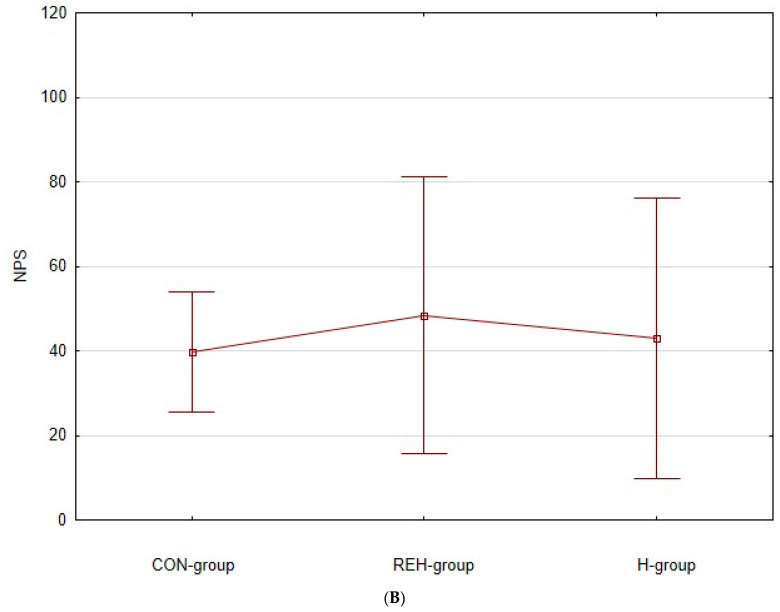
(**A**). NPS initial results: scatterplot CON group versus REH group versus H group. (**B**). NPS initial results means and standard deviations REH group versus CON group versus H group.

As it can be seen from Table 1, all significant study parameters were not statistically different at baseline: PANSS total, PANSS positive, PANSS negative, PANSS general, age at the first hospitalization, NPS serum level, BMI, and age of participants. A scatterplot of NPS initial results REH group versus CON group with the specification of the confidence interval 0.95 is presented in Figure 1.

The patients from the CON group had on average three previous psychiatric hospitalizations (M 2.77, SD 1.60), and the REH group had four (M 4.19, SD 1.17). Almost all the patients lived on a disability pension or other social benefits. A significant proportion of the study participants smoked cigarettes: CON—76.5%; REH—56.3%; non-clinical—66.7%.

During the trial, all patients continued their former anti-psychotic treatment (daily dose olanzapine equivalents in milligrams: CON vs. REH: M 19.32 SD 4.97 vs. M 21.28 SD 6.88) [37]. The anti-psychotic treatment pattern was not changed during the experiment. All subjects were administered atypical anti-psychotics (olanzapine, clozapine, quetiapine, risperidone, and aripiprazole), and only some of them additionally received typical ones (sulpiride, perazine, zuclopenthixol, fluanxol, and haloperidol, respectively: CON—11.8%; REH—20.0%). None of the patients had taken anti-cholinergic drugs. The monotherapy involved an average of half of the study participants: CON group—47.1%; REH group—56.3%.

### 2.3. Outcome Measures

The examinations were performed twice, at the beginning (T1) and after a period of 3 months (T2).

#### 2.3.1. PANSS

Clinical parameters were examined with the positive and negative syndrome scale (PANSS), which is the gold standard for measuring symptoms, syndromes, and the general severity of schizophrenia [38]. Paradoxically, the authors of PANSS themselves tried to modify the scale—restricted initially to the 2-factor model of the disease, with the addition of the so-called general symptoms—converting the scale to fit a multi-factorial model [39]. These further PANSS variants were not only aimed at greater statistical accuracy and reliability of the clinical observations, but also the state of knowledge about schizophrenia could no longer be restricted to the positive–negative concept. It was concluded that these multi-functional models of schizophrenia characterized patients with greater relevance. The needs to go beyond the scheme of 2-factor schizophrenia (positive/productive and negative/deficit symptoms) also resulted from breakthroughs involving new atypical anti-psychotics that affect a much wider spectrum of symptoms and more complex neurochemical mechanisms [40].

However, due to a limited number of participants in this study, any valid factor analysis could not be conducted to fit the structure of PANSS. Therefore, the PANSS results were analyzed using five classical models elaborated by others:

- Two-factor/three-component model; PANSS for typological and dimensional evaluation; consistent with the original PANSS scheme, including positive, negative, and collection of general symptoms [38].
- Four-factor models:
  1. The pyramidical model (triangular pyramid-shaped model); PANSS data redesigned by their own authors; the model included positive, negative, depressive, and excitement factors [39].
  2. The male-specific model derived from the gender-specific PANSS-related trial (structure of PANSS separate for women and men); it consists of positive, negative, cognitive, and hostility factors [35].
- Five-factor models:
  3. The largest multi-ethnic PANSS-related trial (*n* = 3511), validated with the largest meta-analysis; the model of positive, negative, cognitive/disorganization, depression/anxiety, and hostility factors (PANSS factor structure from a large multi-ethnic sample) [41].
  4. The largest pooled data pharmacological analysis, related methodologically to one single anti-psychotic (*n* = 3580); the model of positive, negative, depression/anxiety, cognitive, and excitement/hostility factors (a 5-factor analysis to evaluate the efficacy of iloperidone compared to placebo) [40].

#### 2.3.2. Rehabilitation Therapy

The rehabilitation program in the REH group aimed at changing the daily routine by means of additional social activities, building team competences, training social roles, increasing personal acceptance, and strengthening one’s independence. Structured activities were held for 8 h blocks daily (except at weekends). The general plan of the day included group activities such as psychotherapy, psychoeducation, cognitive therapy, art therapy, physiotherapy, sports, social training, cooking meals together, entertainment activities, and relaxation training. At least one session of psychotherapy or psychoeducation was held every day. The cognitive training included additional neurofeedback sessions throughout the whole 3-month period. We followed the neurofeedback methods of Markiewicz et al. (2020) [42] and Markiewicz et al. (2021) [43]. The forms of active rehabilitation (concerning the REH group) were not “mutually interchangeable” in the time schedule. These were different classes, e.g., aimed at the rehabilitation of cognitive functions, building group cooperation, or acquiring social competences. Part of their effectiveness was their predictability, i.e., each activity had to be announced, discussed, prepared, performed, gratified, and finalized with conclusions. They were also progressive and unique at a given stage of rehabilitation. On the other hand, in the control group (CON), only passive support was provided, without organizing the daily schedule.

#### 2.3.3. Laboratory

The serum level of NPS was determined immunoenzymatically with the ELISA technique (Human NPS/Neuropeptide S ELISA Kit, EIAab Science Co, 6618h catalog number). The NPS level was determined at 07:00 AM (pg/mL), using a non-contact method of blood sampling into a clot tube. Other metabolic parameters that may demonstrate an association of NPS with metabolism (glycemia, total cholesterol, HDL, LDL, triglycerides, AspAT, and AlAT) were also examined from blood samples. Metabolic disorders in patients with schizophrenia—either inherently related to the disease or secondary to adverse effects of anti-psychotic treatment—are so widespread and detrimental that virtually all research into treatment modalities of people with schizophrenia control at least basic biochemical parameters. However, the relationship of NPS and metabolic changes may be much deeper, as a negative correlation of NPS concentration in serum versus BMI was revealed [17]. For these reasons, we performed a screening of some biochemical parameters.

### 2.4. Statistical Analyses

The values of the investigated variables were presented as means and standard deviations. The sociological and demographic parameters were presented as numbers and percentages. The results were compared using Student’s *t*-test for dependent samples, non-parametric Mann–Whitney U-test, and Pearson’s r product–moment correlation coefficient. The Shapiro–Wilk test was used to check whether samples came from a normal distribution. Differences were statistically significant at *p* < 0.05. Analyses were performed using Statistica 13.3.

### 2.5. Ethical Issues

The study protocol was approved by the local Bioethics Committee—approval no. KE-0254/35/2016. All the patients invited to take part in the study gave their written informed consent.

## 3. Results

### 3.1. Long-Term Therapy Results

The baseline versus 3-month results of the rehabilitation group (REH) and control group (CON) are presented in Table 2—PANSS results—and Table 3—NPS and other metabolic results.

There were no significant differences in the PANSS total scores in the REH and CON groups after the 3-month treatment (Table 2. However, based on the five PANSS factor models, some improvements were identified, almost exclusively in the REH group, in terms of: positive factor—Kay et al. [38], Walsh et al. [35], Lim et al. [41], and Citrome et al. [40]; depression/anxiety factor—Kay et al. [39], Lim et al. [41], and Citrome et al. [40]; excitement/hostility factor—Kay et al. [39]. Contrary to this, the CON group worsened in the excitement/hostility factor across nearly all models [39,40,41].

The serum NPS level (Table 3 significantly decreased in the REH group over the course of the 3-month therapy. No significant NPS changes were found in the CON group. Almost no metabolic parameters changed during the entire trial. Only AspAT increased in the REH group. 

### 3.2. Structured Rehabilitation vs. Unstructured Therapy

The REH and CON results were compared for the magnitude of changes of pre- and post-therapy results (T2–T1 differences). 

Changes that occurred over time from T1 to T2 (Table 4, including each of the PANSS models, differentiated significantly for the REH vs. CON 3-month results. These effects were expressed in every PANSS model and nearly all dimensions, clearly illustrating the potential of rehabilitation therapy.

At the same time, the serum NPS level decreased significantly more in the REH versus CON group. Other metabolic effects were different only to a limited extent; body weight, BMI, and AspAT increased in the REH group. Searching for possible relationships with key study results, post hoc correlations of the increase in the AspAT level with the reduction in symptoms of excitement/hostility factors were performed. In the scope of each of the four models, the correlations of changes in AspAT and the corresponding factors were consistently insignificant and very weak/weak in terms of strength (Person’s r, respectively, −0.08 for Kay et al. [39], −0.14 for Walsh et al. [35], −0.23 for Lim et al. [41], and −0.22 for Citrome et al. [40]) [44].

### 3.3. Integrating PANNS and NPS Results 

In order to determine the quantitative and qualitative relationships between clinical results and the serum level of NPS in the group of patients receiving rehabilitation therapy, all four PANSS factor models and pre-existing and final NPS outcomes were analyzed.

**Figure 2:**
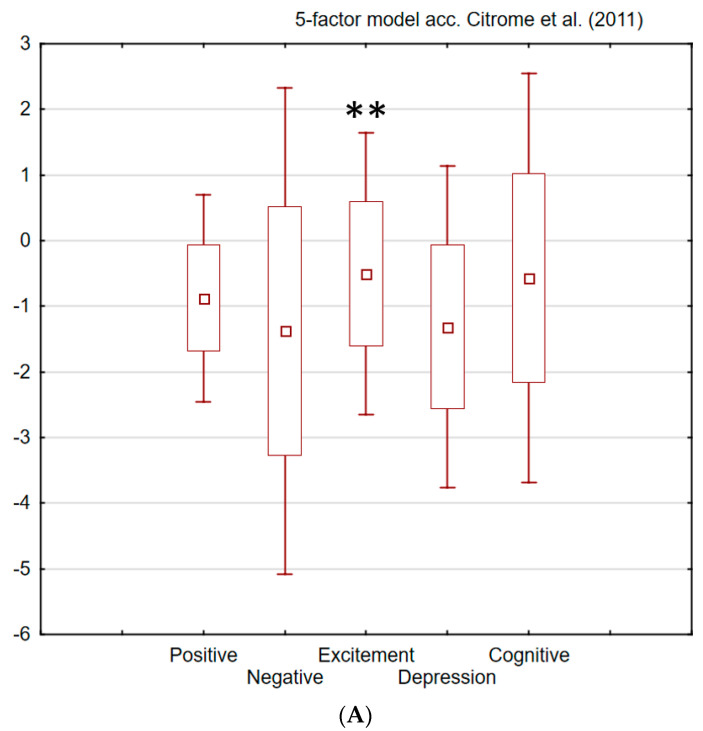

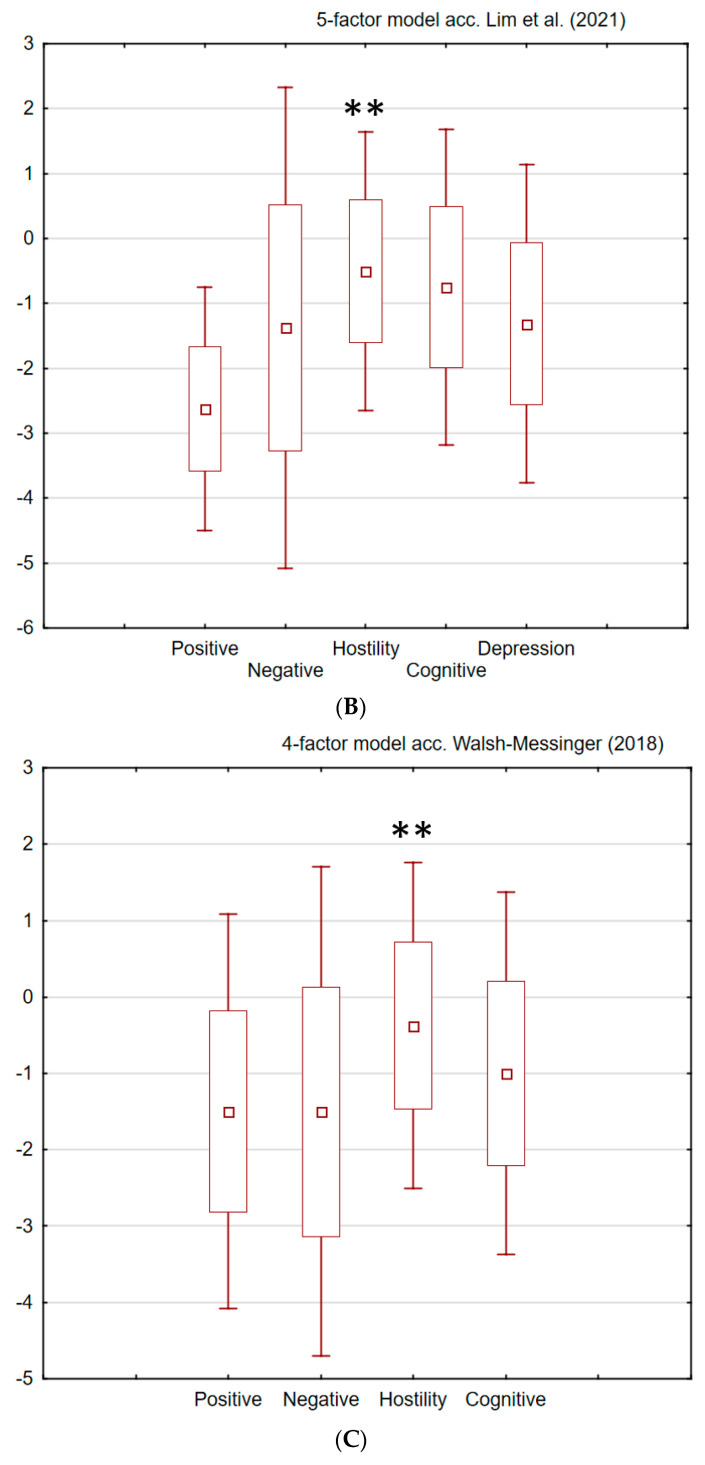

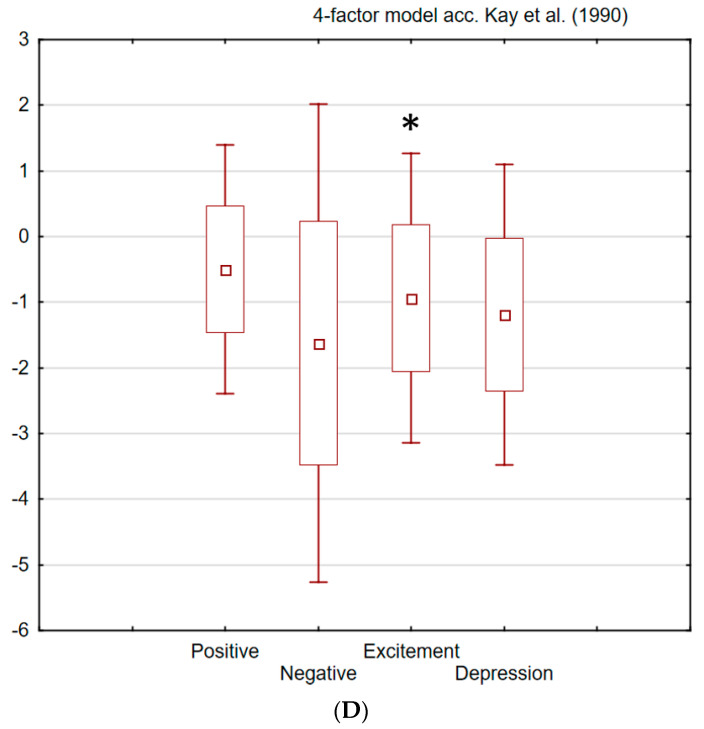
PANSS 3-month rehabilitation therapy results (mean differences for REH group over T1–T2 period) analyzed by four factor models. Factors significantly (*p* < 0.05) and strongly (r > 0.5) correlated with NPS serum level marked with * for T1 and ** for T2. (**A**). Five-factor model according to Citrome et al. (2011) [40]. (**B**). Five-factor model according to Lim et al. (2021) [41]. (**C**). Four-factor model according to Walsh-Messinger et al. (2018) [35]. (**D**). Four-factor model according to Kay et al. (1990) [39].

The magnitude of changes from pre- to post-therapy clinical results in the REH group was correlated selectively and strongly with only one factor, i.e., excitement/hostility (E/H), across all four models (Figure 2). The Pearson’s product–moment correlation (r) is an index of the linear relationship between variables and can be understood as the explanation of the total variability. The correlation analysis was assumed only for the strong relationships, i.e., for absolute values r > 0.5 [44]. 

The most consequently expressed pattern of clinical and biochemical correlations in the REH group across nearly all (3/4) factor models (Walsh-Messinger et al. [35], Citrome et al. [40], and Lim et al. [41]) was the reduction in H/E at T2, accompanied by a decrease in the NPS level at T2. The higher the level of the pre-treatment H/E symptomatology, the more prominent the final reduction in the NPS level that was observed. One model (Kay et al. [39]) also showed a strong correlation between the pre-treatment excitement factor and the NPS level at T1. Pre-existing differences in the NPS level—before rehabilitation therapy was started—were statistically insignificant between the REH vs. CON and REH vs. H groups. However, during the 3-month study, the NPS level was significantly reduced only in the REH group, and the magnitude of this reduction became significantly different between the REH and CON groups. The only specific symptomatology accompanying these phenomena was the hostility/excitement factor extracted from PANSS. 

Table 5 shows the correlations of clinical outcomes and NPS, limited only to the relationship for r > 0.5. Therefore, only the model-specific excitement/hostility factors were included. It is worth noting that the reduction in total PANSS was also strongly correlated with the reduction in NPS (r = −0.54).

### 3.4. Pharmacology

The patients’ anti-psychotic treatment schedule was not modified throughout the study. However, while conducting various post hoc analyses, some significant effects related to the types of anti-psychotic therapy were unveiled. In patients treated with dibenzodiazepine anti-psychotics (the olanzapine, clozapine, and quetiapine group) as monotherapy (39% of all therapies) versus all other anti-psychotic schedules, there was a significantly greater reduction in the NPS level (M −10.6 SD 17.1 vs. M 3.6 SD 10.8, U Mann–Whitney 21.0, *p* 0.039) in the combined REH and CON groups. No other anti-psychotic monotherapy or polytherapy schedule had similar or different results.

## 4. Discussion

The use of the structured rehabilitation therapy resulted in an improvement in the clinical parameters of patients with schizophrenia. At the same time, a significant reduction in the serum NPS level was identified. A strong correlation between the excitement/hostility (E/H) factor reduction and decrease in the NPS level during the 3-month rehabilitation therapy was also observed.

Thus far, there have been no clinical studies with the primary goal of assessing the NPS serum level in relation to schizophrenia symptomatology and treatment. The excitement/hostility (E/H) factor seemed to be specifically related to NPS 3-month scores, which was validated in a series of PANSS factor models. 

Considering the original PANSS model [38], a significant correlation in NPS T1, NPS T2, and NPS T2–T1 was affiliated only to general and total PANSS results, but not to its primary positive and negative factors. The reduction in symptoms in the general subscale was correlated with the NPS decrease; however, since the general subscale is a collection of a variety of symptoms, the result could not be tracked further based on the original PANSS model.

A modified four-factor model [39] revealed the specificity of the relationship. The decreasing NPS level correlated with the excitement factor, grouping symptoms of psychotic disorganization such as poor impulse control, tension, hostility, and uncooperativeness.

Using a narrowly defined gender model developed specifically for males [35], this effect was also confirmed. The decrease in the NPS serum level correlated strongly with the decrease in the severity of symptoms of the hostility factor, accompanied by a lack of judgment and insight.

In two other models, derived from the largest PANSS trials ever conducted [40,41], the NPS level was still specifically and selectively associated with the hostility factor [40] or its excitement/hostility variant [40].

Contrary to this, considering all PANSS models [35,38,39,40,41], there was no significant correlation between NPS and any other clinical factor (i.e., positive, negative, cognitive, or depression/anxiety). When assessing the strength of this relationship, it should be noted that the main effect of the E/H factor alone produced a significant correlation in the PANSS total T2–T1 result with NPS T2–T1.

A review of the NPS correlations with the PANSS models indicated that the lower the NPS scores in both T1 and T2, the lower the psychotic disorganization that was observed; therefore, proportionally, the greater the NPS decrease between T1 and T2 (3-month period), the greater the clinical improvement that could be expected within the range defined by the reduction in the excitement/hostility symptoms. Our study exposed the diagnostic, prognostic, and therapeutic potential of NPS screening in patients with schizophrenia, especially in relation to the E/H domain. Generally, E/H symptoms reduction can be associated with the core impact of rehabilitation, which is anxiety reduction, which is related to the overall research on NPS [6,45,46]. The results were so specific and selective, repeated across various PANSS models, that NPS may be understood as the marker of excitement/Hostility symptomatology. The activities of the NPS/NPSR system were consistent in many aspects with theories explaining the psychotic disorganization related to the E/H factor [47]. Moreover, in animal studies, NPS can reduce the stress reaction, and stress is virtually the mandatory element of all vulnerability concepts of schizophrenia [2,7,48]. NPS can alleviate neuropathological, neurochemical, and behavioral effects produced by NMDA receptor antagonists, which is conceptualized as the model of schizophrenia in animal studies [26]. Animals pre-treated with NPS are protected against the MK-801-induced disruption in cognitive pre-pulse inhibition. NPS also has the potential for immune regulation, and it is known that alleviating inflammation in people with schizophrenia can provide therapeutic benefits, regardless of the cause [9,14,17,49]. The NPS central activity was highlighted as the model of ‘arousal without anxiety’, which gives hope for the ability to improve fundamental deficits in schizophrenia, often even further exacerbated by using sedative anti-psychotics and anti-cholinergic drugs [50]. Finally, NPS is directly related to the most classic dopaminergic hypothesis of schizophrenia through stimulating dopamine release [3]. An experimental 7-day NPS administration to substantia nigra protected dopaminergic neurons from degeneration, possibly by reducing oxidative damage to lipids and proteins.

Central and peripheral NPS expression must be compared and analyzed with caution, considering the multi-directional nature of NPS activities, as well as the short- and long-term diversification [27,28,51,52,53]. Our active group benefited from long-term rehabilitation and, like other cognitive and/or behavioral training, was focused on long-term brain neuroplasticity [54]. The issue of NPS specificity for rehabilitation therapy and/or pharmacotherapy is inconclusive; our primary results were correlated with rehabilitation; however, some indirect relation with dibenzodiazepine anti-psychotics monotherapy was also identified. Possibly, our results and those of basic pharmacological studies may, in fact, be complementary [27,28]. As has been proven, the blood–brain barrier permeability can be studied indirectly through biomarkers in blood [55]. Serum NPS levels have already been used as a marker of mental disorders [46]. We know also from animal studies that NPS penetrates the BBB quite easily, causing biological effects [56]. Changes in serum NPS levels should not be interpreted only mechanistically or statistically, but in the context of an increasing body of research on blood–brain barrier (BBB) dysfunction in schizophrenia, leading to central neuroinflammation [57,58,59].

The decrease in NPS over the 3-month study period did not correlate with many basic metabolic measurements. The NPS T2 results and the NPS T2–T1 difference were not related to the body mass index (BMI), total cholesterol, HDL, LDL, triglycerides, glycemia, and AlAT. The role of NPS/NPSR1 in food intake and metabolic regulation is not clear, and our trial did not support any of the anabolic or anorectic hypotheses [16,17].

A significant increase in the level of AspAT in the REH group and, consequently, a difference in the level between the CON and REH groups after the 3-month treatment period was not correlated with any of the key clinical results, i.e., it was insignificant for the reduction in symptoms of excitement/hostility, across all four factor models. The increase in AspAT itself was moderate, and this effect is typically observed in the case of the use of dibenzodiazepine anti-psychotics, which were also administered in this study [60].

Our present study validated the NPS serum level as a promising target for the long-term clinical evaluation of the effectiveness of schizophrenia therapy. The specific relationship of NPS with a selected group of disorganization symptoms (excitement, hostility) makes it possible to conduct a more targeted anti-psychotic therapy. The results encourage further research on the relationship between NPS and the development of schizophrenia itself, as well as its treatment in general [61]. However, the study had some important limitations (small groups, males only, episodic schizophrenia sub-type only, and mixed rehabilitation/pharmacological therapy), so the verification of all hypotheses requires extending the current study. Some animal studies may support the role of NPS in this regard, but we could not use them directly as the major limitation in animal research is the lack of a comprehensive schizophrenia model considering all aspects of human psychosis. The results of the work require confirmation in larger groups, but they are nevertheless pioneering and open the field for multi-directional research, in particular psychopharmacogenetic and molecular studies. It is crucial to integrate future results with genuinely unmet therapeutic targets, as in the case for schizophrenia.

## 5. Conclusions

1. The structured rehabilitation therapy compared to the unstructured supportive therapy significantly reduced the level of schizophrenia disorders defined by various factor models derived from the PANSS.
2. The clinical improvement within the 3-month rehabilitation therapy course was correlated with a significant decrease in the neuropeptide S (NPS) serum level.
3. The excitement/hostility (E/H) factor, which included schizophrenic symptoms of psychotic disorganization, was specific and selective for the reduction in serum NPS, which was stable across all analyzed factor models.
4. The long-term relationship between serum NPS and clinical factors was not accompanied by changes in basic metabolic parameters.

## Figures and Tables

**Table 1:** Initial (T1) parameters and results for REH, CON, and non-clinical groups.

Variable	REH		CON		REH vs. CON		Non-Clinical (H)		REH vs. Non-Clinical (H)	
	M	SD	M	SD	t ^t^/U ^U^	*p*	M	SD	t ^t^/U ^U^	*p*
PANSS total	53.13	7.29	53.41	15.73	119.0 ^U^	0.552				
PANSS positive	9.75	1.73	10.00	2.40	134.5 ^U^	0.971				
PANSS negative	15.44	3.46	15.29	3.64	−0.12 ^t^	0.909				
PANSS general	27.94	3.55	28.12	10.83	130.5 ^U^	0.857				
Age of first hospitalization (years)	22.69	3.36	25.12	5.10	1.61 ^t^	0.119				
Anti-psychotics in milligrams (equivalents of olanzapine)	21.28	6.88	19.32	4.97	121.5 ^U^	0.614				
NPS (pg/mL)	48.46	16.32	39.67	7.14	82.5 ^U^	0.061	42.97	16.55	64.0 ^U^	0.360
BMI (kg/m^2)^	29.84	4.05	27.39	2.81	−2.02 ^t^	0.052	28.85	3.88	0.69 ^t^	0.496
Age (years)	36.00	7.79	39.35	10.65	1.03 ^t^	0.312	41.27	7.48	−1.92 ^t^	0.065

REH—patient rehabilitation group; CON—patient control group; non-clinical—healthy (H) reference group; PANSS—positive and negative syndrome scale; PANSS total—total result of PANSS; PANSS positive—subscale of positive symptoms of PANSS; PANSS negative—subscale of negative symptoms of PANSS; PANSS general—subscale of general symptoms of PANSS; NPS—neuropeptide S; BMI—body mass index; M—mean; SD—standard deviation; ^t^—Student’s *t*-test; ^U^—Mann–Whitney U-test; *p*—*p*-value significance at *p* < 0.05.

**Table 2:** T1 versus T2 results: PANSS models and factors.

Model	Factor	Group	Baseline		Final		t	*p*
			M	SD	M	SD		
Kay et al. (1987)	Total	REH	53.13	7.29	48.50	8.22	−1.68	0.103
		CON	53.41	15.73	57.88	7.40	1.06	0.297
	Positive	REH	9.75	1.73	8.25	1.39	−2.70	**0.011**
		CON	10.00	2.40	9.88	8.25	−0.12	0.906
	Negative	REH	15.44	3.46	14.00	3.39	−1.19	0.2445
		CON	15.29	3.64	16.65	2.71	1.23	0.228
	General	REH	27.94	3.55	26.25	4.51	−1.18	0.2445
		CON	28.12	10.83	31.35	3.20	1.18	0.246
Kay et al. (1990)	Positive	REH	7.63	1.09	7.13	1.15	−1.27	0.216
		CON	7.65	3.12	7.24	1.68	−0.48	0.635
	Negative	REH	19.75	3.47	18.13	3.70	−1.28	0.210
		CON	19.41	6.41	21.47	3.22	1.18	0.246
	Depressive	REH	9.19	1.17	8.00	1.97	−2.08	**0.046**
		CON	9.12	2.76	10.18	1.19	1.45	0.156
	Excitement	REH	7.88	1.26	6.94	1.12	−2.22	**0.034**
		CON	7.18	1.88	8.76	1.75	2.55	**0.016**
Walsh-Messinger et al. (2018)	Positive	REH	10.13	1.75	8.63	2.03	−2.24	**0.033**
		CON	9.35	2.87	10.06	1.75	0.87	0.393
	Negative	REH	13.81	2.74	12.31	3.07	−1.46	0.155
		CON	13.53	4.35	14.71	2.42	0.98	0.337
	Cognitive	REH	13.19	1.94	12.19	2.40	−1.30	0.205
		CON	14.47	4.69	15.41	2.60	0.72	0.475
	Hostility	REH	7.69	1.30	7.31	1.14	−0.87	0.393
		CON	7.24	2.51	8.41	1.80	1.57	0.127
Lim et al. (2021)	Positive	REH	9.25	1.34	6.63	1.20	−5.82	**0.000**
		CON	9.12	3.62	6.94	1.82	−2.22	**0.034**
	Negative	REH	14.25	2.84	12.88	3.10	−1.31	0.200
		CON	13.82	4.17	15.53	2.43	1.46	0.155
	Cognitive	REH	13.56	2.34	12.81	2.46	−0.88	0.383
		CON	15.18	4.93	15.29	2.76	0.09	0.932
	Depression/Anxiety	REH	9.00	1.21	7.69	1.96	−2.28	**0.030**
		CON	8.59	2.62	9.88	1.41	1.79	0.083
	Hostility	REH	5.94	1.18	5.44	0.81	−1.39	0.174
		CON	5.29	1.53	6.71	1.53	2.69	**0.011**
Citrome et al. (2011)	Positive	REH	7.50	1.15	6.63	1.20	−2.10	**0.044**
		CON	7.24	2.51	6.94	1.82	−0.39	0.699
	Negative	REH	14.25	2.84	12.88	3.10	−1.31	0.200
		CON	13.82	4.17	15.53	2.43	1.46	0.155
	Depression/Anxiety	REH	9.00	1.21	7.69	1.96	−2.28	**0.030**
		CON	8.59	2.62	9.88	1.41	1.79	0.083
	Cognitive	REH	16.44	2.73	15.88	3.07	−0.55	0.588
		CON	18.47	6.28	18.82	3.19	0.21	0.838
	Excitement/Hostility	REH	5.94	1.18	5.44	0.81	−1.39	0.174
		CON	5.29	1.53	6.71	1.53	2.69	**0.011**

REH—patient rehabilitation group; CON—patient control group; PANSS—positive and negative syndrome scale; PANSS clinical factors or subscales as defined by models: anxiety, cognitive, depressive/depression, disorganization, excitement, general, hostility, positive, and negative; M—mean; SD—standard deviation; t—Student’s *t*-test; *p*—*p*-value significance at *p* < 0.05.

**Table 3:** T1 versus T2 results: NPS and metabolic parameters.

Variable	Group	Baseline		Final		t	*p*
		M	SD	M	SD		
NPS (pg/mL)	REH	48.46	16.32	36.01	3.45	−2.99	**0.006**
	CON	39.67	7.14	38.96	6.76	−0.30	0.766
Body mass (kg)	REH	95.50	13.92	97.06	14.09	0.34	0.736
	CON	88.18	12.39	85.50	13.40	−0.61	0.550
BMI (kg/m^2^)	REH	29.84	4.05	30.33	4.23	0.34	0.736
	CON	27.39	2.81	26.54	3.09	−0.84	0.408
Cholesterol total (mg/dL)	REH	216.39	46.34	221.19	200.90	0.32	0.758
	CON	196.48	41.35	200.90	26.41	0.37	0.713
HDL (mg/dL)	REH	42.00	5.94	41.94	5.09	−0.03	0.975
	CON	39.69	8.13	38.91	6.09	−0.32	0.755
LDL (mg/dL)	REH	97.00	18.30	98.88	16.52	0.30	0.763
	CON	111.24	25.40	104.94	22.00	−0.77	0.446
Triglycerides (mg/dL)	REH	117.25	30.13	119.69	38.28	0.20	0.843
	CON	121.88	44.28	148.59	64.56	1.41	0.169
Glycaemia (mg/dL)	REH	91.88	9.51	98.50	15.41	1.46	0.154
	CON	86.41	11.66	90.82	14.54	0.98	0.336
AlAT (IU/L)	REH	30.50	14.80	37.44	30.69	0.81	0.422
	CON	29.66	18.11	30.19	9.77	0.11	0.915
AspAT (IU/L)	REH	21.99	5.25	32.14	16.78	2.33	**0.027**
	CON	24.42	8.23	26.11	7.70	0.62	0.541

REH—patient rehabilitation group; CON—patient control group; PANSS factors or subscales as defined by models: anxiety, cognitive, depressive/depression, disorganization, excitement, general, hostility, positive, and negative; NPS—neuropeptide S; BMI—body mass index; HDL—high-density lipoprotein; LDL—low-density lipoprotein; AlAT—alanine transaminase; AspAT—aspartate transaminase; M—mean; SD—standard deviation; t—Student’s *t*-test; *p*—*p*-value significance at *p* < 0.05.

**Table 4:** Differences in the magnitude of changes from pre- (T1) to post-therapy (T2) results between REH and CON groups.

Variable		REH (T2–T1)		CON (T2–T1)		In-Between Comparisons	
		M	SD	M	SD	t/U	*p*
PANSS for typological and dimensional assessment [37]	Total	−4.63	3.40	4.47	10.93	23.50 ^U^	**0.000**
	Positive	−1.50	1.26	−0.12	1.32	59.50 ^U^	**0.006**
	Negative	−1.44	1.46	1.35	3.06	3.31 ^t^	**0.002**
	General	−1.69	2.02	3.24	9.08	23.00 ^U^	**0.000**
Pyramid-like triangular model [38]	Positive	−0.50	0.97	−0.41	1.84	94.00 ^U^	**0.140**
	Negative	−1.63	1.86	2.06	5.44	29.00 ^U^	**0.000**
	Depressive	−1.19	1.17	1.06	1.18	3.18 ^t^	**0.003**
	Excitement	−0.94	1.12	1.59	2.59	6.30 ^t^	**0.000**
Structure of PANSS separately in males and females [34]	Positive	−1.50	1.32	0.71	1.61	4.29 ^t^	**0.000**
	Negative	−1.50	1.63	1.18	3.64	2.69 ^t^	**0.011**
	Cognitive	−1.00	1.21	0.94	3.49	50.00 ^U^	**0.002**
	Hostility	−0.38	1.09	1.18	1.47	3.43 ^t^	**0.002**
PANSS factor structure from a large multi-ethnic sample [40]	Positive	−2.63	0.96	−2.18	2.04	82.50 ^U^	0.056
	Negative	−1.38	1.89	1.71	3.62	3.04 ^t^	**0.005**
	Cognitive	−0.75	0.12	1.24	3.76	74.00 ^U^	**0.027**
	Depression/anxiety	−1.31	1.25	1.29	2.59	3.65 ^t^	**0.001**
	Hostility	−0.50	1.10	1.41	1.00	5.23 ^t^	**0.000**
Five-factor analysis for evaluating the efficacy of iloperidone vs. placebo [39]	Positive	−0.88	0.81	−0.29	0.92	72.00 ^U^	**0.022**
	Negative	−1.38	1.89	1.71	3.62	3.04 ^t^	**0.005**
	Depression/anxiety	−1.31	1.25	1.29	2.59	3.64 ^t^	**0.001**
	Cognitive	−0.56	1.59	0.35	4.81	71.00 ^U^	**0.020**
	Excitement/hostility	−0.50	1.10	1.41	1.00	5.23 ^t^	**0.000**
NPS (pg/mL)		−12.46	15.97	−0.72	9.97	71.00 ^U^	**0.020**
Body mass (kg)		1.56	2.03	−2.68	3.76	38.50 ^U^	**0.001**
BMI (kg/m^2^)		0.50	0.66	−0.85	1.13	32.50 ^U^	**0.000**
Cholesterol total (mg/dL)		4.80	21.51	4.42	46.08	−0.03 ^t^	0.977
HDL (mg/dL)		−0.06	2.74	−0.78	5.92	128.00 ^U^	0.787
LDL (mg/dL)		1.88	5.93	−6.29	24.53	105.50 ^U^	0.279
Triglycerides (mg/dL)		2.44	13.89	26.71	70.20	111.00 ^U^	0.378
Glycaemia (mg/dL)		6.63	11.68	4.42	15.44	135.00 ^U^	0.986
AlAT (IU/mL)		6.94	23.24	0.54	10.85	127.00 ^U^	0.760
AspAT (IU/mL)		10.24	15.78	1.69	5.32	74.50 ^U^	**0.028**

REH—patient rehabilitation group; CON—patient control group; PANSS—positive and negative syndrome scale; PANSS factors or subscales as defined by models: anxiety, cognitive, depressive/depression, disorganization, excitement, general, hostility, positive, and negative; NPS—neuropeptide S; BMI—body mass index; HDL—high-density lipoprotein; LDL—low-density lipoprotein; AlAT—alanine transaminase; AspAT—aspartate transaminase; M—mean; SD—standard deviation; ^t^—Student’s *t*-test; ^U^—Mann–Whitney U-test; *p*—*p*-value significance at *p* < 0.05 (bold).

**Table 5:** The Pearson’s r product–moment correlation coefficients for REH group: NPS T1, NPS T2, and NPS T2–T1 correlated with excitement/hostility factors. Strong correlations for absolute values of r > 0.5 (*p* < 0.05) were bolded.

Model	Factor		NPS T1	NPS T2	NPS T2–T1
Kay et al. (1990)	Excitement	T1	**−0.54**	0.40	**0.64**
		T2	−0.34	−0.05	0.34
		T2–T1	0.29	−0.49	−0.41
Walsh-Messinger et al. (2018)	Hostility	T1	−0.50	0.44	**0.61**
		T2	−0.40	−0.01	0.40
		T2–T1	0.27	**−0.55**	−0.40
Lim et al. (2021)	Hostility	T1	−0.50	0.44	**0.61**
		T2	−0.47	−0.22	0.44
		T2–T1	0.22	**−0.61**	−0.36
Citrome et al. (2011)	Excitement/hostility	T1	−0.50	0.44	**0.61**
		T2	−0.47	−0.22	0.44
		T2–T1	0.22	**−0.61**	−0.36

REH—rehabilitation therapy group; T1—pre-therapy results; T2—post-therapy results; NPS—neuropeptide S.

## Data Availability

Not applicable.
